# Variational Bayesian Innovation Saturation Kalman Filter for Micro-Electro-Mechanical System–Inertial Navigation System/Polarization Compass Integrated Navigation

**DOI:** 10.3390/mi16091036

**Published:** 2025-09-10

**Authors:** Yu Sun, Xiaojie Liu, Xiaochen Liu, Huijun Zhao, Chenguang Wang, Huiliang Cao, Chong Shen

**Affiliations:** Key Laboratory of Instrumentation Science & Dynamic Measurement, Ministry of Education, School of Instruments and Electronics, North University of China, Taiyuan 030051, China; sz202306152@st.nuc.edu.cn (Y.S.); b20220636@st.nuc.edu.cn (X.L.); liuxiaochen@nuc.edu.cn (X.L.); zhaohuijun@nuc.edu.cn (H.Z.); caohuiliang@nuc.edu.cn (H.C.); shenchong@nuc.edu.cn (C.S.)

**Keywords:** variational Bayesian, integrated navigation system, Student’s *t*-distribution, Kalman filter, polarization compass

## Abstract

Aiming at the issue of time-varying measurement noise with heavy-tailed characteristics and outliers generated by the polarization compass (PC) in the micro-electro-mechanical system–inertial navigation system (MEMS-INS) and PC-integrated navigation system when it is subject to internal and external disturbances, an improved Variational Bayesian Innovation Saturation Robust Adaptive Kalman filter (VISKF) algorithm is proposed. This algorithm utilizes the variational Bayesian (VB) method based on Student’s *t*-distribution (STD) to approximately calculate the statistical characteristics of the time-varying measurement noise of the PC, thereby obtaining more accurate measurement noise statistical parameters. Additionally, the algorithm introduces an innovation saturation function and proposes an adaptive update strategy for the saturation boundary. It mitigates the problem of innovation value divergence in PC caused by outliers through a two-layer structure that can track the changes in the innovation value to adaptively adjust the saturation boundary. To verify the effectiveness of the algorithm, static and dynamic experiments were conducted on an unmanned vehicle. The experimental results show that compared with adaptive Kalman filter (AKF), variational Bayesian robust adaptive Kalman filter (VBRAKF), and innovation saturate robust adaptive Kalman filter (ISRAKF), the proposed algorithm improves the dynamic orientation accuracy by 76.89%, 67.23%, and 84.45%, respectively. Moreover, compared with other similar target algorithms, the proposed algorithm also has obvious advantages. Therefore, this method can significantly improve the navigation accuracy and robustness of the INS/PC integrated navigation system in complex environments.

## 1. Introduction

Autonomous navigation is the crucial technology for automating and intelligent vehicles, with extensive applications in space exploration, aviation, maritime transportation, ground vehicles [1]. Currently, the micro-electro-mechanical system–inertial navigation system (MEMS-INS) and Global Navigation Satellite System (GNSS) are the most widely employed integrated navigation methods for acquiring navigation information [2,3,4,5]. However, INS errors accumulate over time [6,7,8]. Meanwhile, GNSS, which relies on satellite signals from space, is susceptible to both natural and man-made interference, resulting in reduced accuracy [9,10]. Therefore, bionic polarization navigation technology has emerged as a promising alternative due to its advantages in independence, concealment, continuity, autonomy, resistance to electromagnetic interference, and high precision [11]. This technology leverages the polarization characteristics of light to detect the polarization distribution patterns of sky or sunlight, thereby obtaining the heading angle information of the carrier [9]. Extensive research has demonstrated that the novel autonomous navigation system combining MEMS-INS and polarization compass (PC) exhibits superior performance, effectively meeting the stringent requirements for high-precision and high-robustness navigation information acquisition.

Kalman filtering is a widely adopted method for fusing PC and MEMS-INS information. However, its performance critically depends on the accurate modeling of the random characteristics of both system processes and measurement noise [12]. In complex scenarios—such as lens occlusion or high-speed carrier maneuvers—the measurement noise of the PC exhibits time-varying and heavy-tailed characteristics [13,14], introducing stochastic and systematic errors into system modeling and data acquisition that substantially degrade navigation accuracy [1]. These factors contribute to measurement uncertainty in the PC, significantly undermining the performance of the INS/PC integrated navigation system.

To address measurement uncertainty and enhance the robustness of integrated navigation systems, extensive research has been conducted on robust Kalman filtering and adaptive filtering algorithms. In the realm of robust Kalman filtering, methods such as the Huber-based Kalman Filter (HKF) [15,16], Maximum Entropy Criterion-based Kalman Filter (MCCKF) [17,18], and Student’s t-distribution (STD)-based Kalman Filter [19,20,21,22] have garnered significant attention. Meanwhile, many adaptive stochastic models directly estimate the covariance matrices of system process and measurement noise, monitor changes in the covariance of innovations and residuals, and make timely adjustments. For instance, Hide et al. [23] developed a covariance scaling method that enhances filter robustness by introducing scaling factors into the covariance matrix. Yang et al. [24] designed a robust adaptive filter that combines robust maximum likelihood estimation with adaptive filtering, adjusting the weights of the predicted parameters based on the differences between system measurements and predicted innovation values. Gao et al. [25] proposed a robust adaptive filter that adaptively adjusts the state noise covariance matrix using an adaptive factor derived from prediction residuals.

Significant research has been conducted on robust adaptive filtering for INS/PC integrated navigation systems. For instance, Zhao et al. [13] proposed an enhanced Variational Bayesian Cubature Kalman Filter (VBCKF) to estimate the time-varying measurement noise covariance of the sub-filters. While this approach can partially capture the variations in measurement noise in complex environments, it simplifies the modeling of measurement noise and does not account for its heavy-tailed characteristics. To address the issue of outliers, Zhao et al. introduced a residual Chi-squared detection algorithm to identify information mismatches or sensor faults, along with a residual-based compensation algorithm to mitigate abnormal PC measurements. This method detects faults using thresholds determined by the Chi-squared distribution; however, it is not sensitive to small outliers. Dou et al. [26] proposed an adaptive adjustment method based on the degree of polarization to tackle the problem of outliers in PC measurements. In their work, the degree of polarization was defined as a deterministic factor to adaptively adjust the measurement noise matrix by modifying the update rule. Although this approach improved the stability of the sky polarization light-based autonomous navigation system under outlier conditions, it only adjusts the measurement noise matrix by constraining the update term with a deterministic factor, failing to accurately capture the variations in measurement noise.

Building on previous research, high-precision and robust INS/PC integrated navigation systems face several key challenges. One significant issue is accurately modeling the time-varying measurement noise of the PC, particularly its heavy-tailed characteristics, which conventional approaches struggle to capture effectively. Additionally, existing INS/PC fusion methods lack the flexibility to adapt the noise covariance matrix in real time, reducing their robustness in dynamic environments. Furthermore, outliers caused by internal sensor errors and external disturbances exacerbate these challenges, as they are not effectively mitigated by current filtering methods. Addressing these limitations is critical to enhancing system accuracy and reliability.

In the field of robust filtering algorithms, Kalman filters based on the STD have recently emerged as a prominent area of research, with the STD-based measurement uncertainty filter already applied in integrated navigation systems for complex underwater environments [27]. Motivated by this approach and in response to the challenges outlined above, this paper proposes a STD-based Variational Bayesian Innovation Saturated Kalman Filter (VISKF).

The primary contributions of this paper can be summarized as follows:

(1) Modeling and estimating the measurement noise of PC in complex environments. This study developed a VB estimation method based on the STD to model and estimate the measurement noise of PC in complex environments. The remodeling of the measurement noise using the STD aims to better capture the heavy-tailed characteristics of the noise in such environments. Subsequently, this study employed VB theory to estimate statistical characteristic parameters of the measurement noise, resulting in a more accurate model.

(2) Innovation saturated the robust adaptive fusion method. This method introduces a predictive innovation saturation (IS) function to address the propagation of innovation errors caused by anomalous PC measurements. To further enhance robustness, an adaptive innovation saturation boundary strategy is employed, ensuring the retention of accurate values while suppressing erroneous ones. The boundary is dynamically adjusted through a dual-layer structure: the lower layer monitors and responds to changes in innovation values, while the upper layer refines the boundary to maintain precision. This design ensures accurate corrections to enhance system performance.

The remainder of this paper is structured as follows: Section 2 provides a comprehensive overview of the modeling of the INS/PC. Section 3 presents detailed insights into the proposed algorithm. Section 4 outlines the experimental process and data analysis results. Finally, in Section 5, a conclusion is drawn and a brief summary of the paper is provided.

## 2. INS/PC Integrated Navigation System Model

The navigation frames (n-frames) referenced in this paper are orthogonal reference frames aligned with the East–North–Up (E-N-U) geodetic axes. The INS/PC integrated navigation system can be described as follows:

(1) $$\left\{\begin{array}{l} \dot{X}=FX+N\\ Z=HX+V \end{array}\right.,$$

where F is the state transition matrix, given by

(2) $$F=\left[\begin{array}{ccccc} M_{aa} & M_{av} & M_{ap} & -C_{b}^{n} & 0_{3\times 3}\\ M_{va} & M_{vv} & M_{vp} & 0_{3\times 3} & C_{b}^{n}\\ 0_{3\times 3} & M_{pv} & M_{pp} & 0_{3\times 3} & 0_{3\times 3}\\ & & 0_{6\times 15} & & \end{array}\right],$$

where $M_{aa}$, $M_{av}$, $M_{ap}$, $M_{va}$, $M_{vv}$, $M_{vp}$, $M_{pv}$, and $M_{pp}$ can be found in [28]. N is the process noise vector, Z signifies the measurement information of the PC, and H denotes the system observation matrix. V is the measurement noise, and X is the state vector, and its representation is as follows:

(3) $$X={\left[\begin{array}{lllll} \phi_{1\times 3}^{T} & \delta V_{1\times 3}^{T} & \delta P_{1\times 3}^{T} & \delta \omega_{1\times 3}^{T} & \delta f_{1\times 3}^{T} \end{array}\right]}^{T},$$

where $\phi_{1\times 3}={\left[\;\begin{array}{ccc} \delta \theta & \delta \gamma & \delta \psi \end{array}\right]}^{T}$ denotes the attitude error, $\delta V_{1\times 3}={\left[\begin{array}{ccc} \delta v_{E}^{n} & \delta v_{N}^{n} & \delta v_{U}^{n} \end{array}\right]}^{T}$ represents the velocity error vector, and $\delta P_{1\times 3}={\left[\begin{array}{ccc} \delta L & \delta \lambda & \delta h \end{array}\right]}^{T}$ indicates the position error vector. $\delta \omega_{1\times 3}={\left[\begin{array}{ccc} \omega_{x}^{b} & \omega_{y}^{b} & \omega_{z}^{b} \end{array}\right]}^{T}$ is the gyroscope drift error vector, and $\delta f_{1\times 3}={\left[\begin{array}{ccc} f_{x}^{b} & f_{y}^{b} & f_{z}^{b} \end{array}\right]}^{T}$ is the accelerometer bias error vector.

Upon discretization of the state equation, the following expression can be derived:

(4) $$x_{k}=\left(I+F\Delta t\right)x_{k-1}+n_{k-1}\Delta t=\Phi_{k-1}x_{k-1}+w_{k-1},$$

where k represents the system instant, Δt denotes the sampling interval, $\Phi_{k-1}$ represents the discrete state transition matrix, and $w_{k-1}$ signifies the discrete process noise matrix.

The following equation can be derived using the heading output obtained from the PC and INS:

(5) $$Z={\left[\begin{array}{cc} Z_{\phi } & 0_{1\times 12} \end{array}\right]}^{T},\;Z_{\phi }=[\begin{array}{ccc} 0 & 0 & \psi_{INS}-\psi_{PC} \end{array}],$$

(6) $$Z_{\phi }=\left(\begin{array}{ccc} 0 & 0 & 0\\ 0 & 0 & 0\\ 0 & 0 & 1 \end{array}\right)\phi =M_{\psi }\phi ,$$

where $M_{\psi }$ represent the observation matrix. $\psi_{INS}$ and $\psi_{PC}$ denote the heading angles produced by INS and PC, respectively. At this point, the discrete representation of the measurement equation can be formulated as follows:

(7) $$z_{k}=Hx_{k}+v_{k},$$

(8) $$H=\left[\begin{array}{llll} M_{\psi } & 0_{3\times 3} & 0_{3\times 3} & 0_{3\times 6}\\ 0_{3\times 3} & I_{3\times 3} & 0_{3\times 3} & 0_{3\times 6}\\ 0_{3\times 3} & 0_{3\times 3} & I_{3\times 3} & 0_{3\times 6} \end{array}\right],$$

where $z_{k}$ represents the difference between the heading angle of PC and INS. H denotes the measurement matrix, while $v_{k}$ signifies the measurement noise of PC. The covariance matrices for process and measurement noise are denoted as $Q_{k}$ and $R_{k}$, respectively.

## 3. VISKF Algorithm

### 3.1. Reformulate a Probabilistic Distribution Model

In the INS/PC integrated system, the INS demonstrates relative stability with few significant outliers, making it reasonable to assume that the process noise follows a Gaussian distribution. As a result, the one-step prediction probability density function (PDF) can be given by

(9) $$p\left(x_{k}|z_{1:k-1}\right)=N\left(x_{k};{\hat{x}}_{k|k-1},P_{k|k-1}\right).$$

The measurement noise $v_{k}$, exhibiting heavy-tailed behavior and following the STD of the PC, can be effectively represented by the following equation:

(10) $$p\left(v_{k}\right)=St\left(v_{k};0,R_{k},\gamma \right),$$

where $St\left(\ldots ;u,a,b\right)$ is the PDF of the STD, where the parameters represent the distribution that satisfies a mean of u, a scale matrix of a, and a degree of freedom of b. Using (10), the heavy-tailed measurement noise of the PC has been modeled as an STD that satisfies a mean of 0, a scale matrix of $R_{k}$, and a degree of freedom of γ.

The PDF of PC measurement likelihood distribution can be delineated as follows:

(11) $$p(z_{k}|x_{k})=St\left(z_{k};H_{k}x_{k},R_{k},\gamma \right).$$

The STD can be interpreted as an infinite mixture of Gaussian distributions [29], $p\left(v_{k}\right)$ and $p(z_{k}|x_{k})$ can be expressed as the convolution of Gaussian and Gamma distributions.

(12) $$p\left(v_{k}\right)=\int N\left(v_{k};0,\frac{R_{k}}{\lambda_{k}}\right)G\left(\lambda_{k};\frac{\gamma }{2},\frac{\gamma }{2}\right)d\lambda_{k},$$

(13) $$p\left(z_{k}|x_{k}\right)=\int N\left(z_{k};H_{k}x_{k},\frac{R_{k}}{\lambda_{k}}\right)G\left(\lambda_{k};\frac{\gamma }{2},\frac{\gamma }{2}\right)d\lambda_{k},$$

where $\lambda_{k}$ is introduced as an auxiliary variable to dynamically adjust the covariance matrix $R_{k}$ of the measurement noise. By expressing the covariance of the measurement noise as $R_{k}/\lambda_{k}$, the model can effectively address heavy-tailed noise, thereby enhancing the accuracy and robustness of the tracking process. By modeling this auxiliary random variable, the distribution characteristics of $R_{k}$ can be indirectly inferred.

The likelihood probability density function of system can be represented in a hierarchical Gaussian structure [30]

(14) $$p\left(z_{k}|x_{k},R_{k},\lambda_{k}\right)=N\left(z_{k};H_{k}x_{k},\frac{R_{k}}{\lambda_{k}}\right),$$

(15) $$\begin{array}{c} p\left(\lambda_{k}\right)=G\left(\lambda_{k};\frac{\gamma }{2},\frac{\gamma }{2}\right)\\ \;=\left(\frac{m+\gamma }{2}-1\right)\log\lambda_{k}-\frac{\lambda_{k}\gamma }{2}-\frac{\lambda_{k}}{2}{\left(z_{k}-H_{k}x_{k}\right)}^{T}R_{k}^{-1}\left(z_{k}-H_{k}x_{k}\right)\\ \;-\frac{1}{2}{\left(x_{k}-{\hat{x}}_{k\left|k-1\right.}\right)}^{T}P_{k\left|k-1\right.}^{-1}\left(x_{k}-{\hat{x}}_{k\left|k-1\right.}\right)+C, \end{array}$$

where C is a constant.

### 3.2. Variational Approximation Estimation

By applying the VB method, each parameter of the PC is treated as an independent variable, enabling us to approximate the joint posterior PDF as follows:

(16) $$p(x_{k},R_{k},\lambda_{k}|z_{1:k})\approx q\left(x_{k}\right)q\left(R_{k}\right)q\left(\lambda_{k}\right).$$

To obtain the solution for the approximate distribution, it is necessary to minimize the Kullback–Leibler Divergence (KLD) between the posterior distribution of the PC measurement information and its approximate distribution. Thus, solving the following equation becomes essential:

(17) $$\begin{array}{c} \left\{q\left(x_{k}\right),q\left(R_{k}\right),q\left(\lambda_{k}\right)\right\}=\\ \arg\min KLD\left(\Theta \left\|p\left(x_{k},R_{k},\lambda_{k}\left|z_{1:k}\right.\right)\right.\right)\left(\Theta =q\left(x_{k}\right),q\left(R_{k}\right),q\left(\lambda_{k}\right)\right), \end{array}$$

where $KLD\left(\cdot \right)$ is used to calculate KLD. Its precise mathematical formulation is given by

(18) $$KLD\left(q\left(z\right)\left\|p\left(z\right)\right.\right)=\int q\left(z\right)\log\frac{q\left(z\right)}{p\left(z\right)}dz.$$

In accordance with the computational approach of VB, expressions for $q\left(x_{k}\right)$, $q\left(R_{k}\right)$, $q\left(\lambda_{k}\right)$ can be derived as

(19) $$\left\{\begin{array}{c} \log q\left(x_{k}\right)=E_{R_{k},\lambda_{k}}\left[\log p\left(x_{k},R_{k},\lambda_{k},z_{1:k}\right)\right]+C_{x}\\ \log q\left(R_{k}\right)=E_{x_{k},\lambda_{k}}\left[\log p\left(x_{k},R_{k},\lambda_{k},z_{1:k}\right)\right]+C_{R}\\ \log q\left(\lambda_{k}\right)=E_{x_{k},R_{k}}\left[\log p\left(x_{k},R_{k},\lambda_{k},z_{1:k}\right)\right]+C_{\lambda }, \end{array}\right.$$

where $C_{x}$, $C_{R}$, and $C_{\lambda }$ are constants. Approximate solutions for the coupled variables are computed by the fixed-point iteration method.

Using the properties of the hierarchical Gaussian state-space model, the joint PDF can be given by

(20) $$\begin{array}{c} p(x_{k},R_{k},\lambda_{k},z_{1:k})\\ =p(z_{k}|x_{k},R_{k},\lambda_{k})p\left(x_{k}|z_{1:k-1}\right)p\left(R_{k}\right)p\left(\lambda_{k}\right)\\ =N\left(z_{k};H_{k}x_{k},\frac{R_{k}}{\lambda_{k}}\right)N\left(x_{k};{\hat{x}}_{k-1},P_{k-1}\right)\times IW\left(R_{k};{\hat{u}}_{k-1},{\hat{U}}_{k-1}\right)G\left(\lambda_{k};\frac{\gamma }{2},\frac{\gamma }{2}\right). \end{array}$$

Using Equations (19) and (20), the following can be obtained:

(21) $$\log q^{\left(n+1\right)}\left(R_{k}\right)=-\frac{1}{2}\left(m+{\hat{u}}_{k-1}+2\right)\log\left|R_{k}\right|-\frac{1}{2}tr\left[{\hat{U}}_{k-1}+E^{\left(n+1\right)}\left[\lambda_{k}\right]A_{k}^{\left(n+1\right)}\right]+C_{R_{k}}.$$

Using Equation (21), $q^{\left(n+1\right)}\left(R_{k}\right)$ is expressed as an inverse Wishart distribution (WD), which can be given by

(22) $$q^{\left(n+1\right)}\left(R_{k}\right)=IW\left(R_{k};{\hat{u}}_{k}^{\left(n+1\right)},{\hat{U}}_{k}^{\left(n+1\right)}\right),$$

where ${\hat{u}}_{k}^{\left(n+1\right)}$ and ${\hat{U}}_{k}^{\left(n+1\right)}$ represent the degrees of freedom and the inverse scale matrix, respectively, which can be given by

(23) $${\hat{u}}_{k|k}^{\left(n+1\right)}={\hat{u}}_{k|k-1}+1,\;{\hat{U}}_{k|k}^{\left(n+1\right)}={\hat{U}}_{k|k-1}+A_{k}^{\left(n+1\right)}E^{\left(n+1\right)}\left[\lambda_{k}\right],$$

where $A_{k}^{\left(n+1\right)}$ is the auxiliary matrix, and it can be given by

(24) $$A_{k}^{\left(n+1\right)}=(z_{k}-H_{k}{\hat{x}}_{k}^{\left(n+1\right)}){\left(z_{k}-H_{k}{\hat{x}}_{k}^{\left(n+1\right)}\right)}^{T}+H_{k}P_{k}^{\left(n+1\right)}H_{k}^{T}.$$

By substituting Equations (15) and (20) into Equation (19), a variational form of $q^{\left(n+1\right)}\left(\lambda_{k}\right)$ can be given by

(25) $$\log q^{\left(n+1\right)}\left(\lambda_{k}\right)=\left(\frac{m+\gamma }{2}-1\right)\log\lambda_{k}-\frac{1}{2}\left(\gamma +tr\left(A_{k}^{\left(n\right)}E^{\left(n\right)}\left[R_{k}^{-1}\right]\right)\right)\lambda_{k}+C_{\lambda_{k}},$$

where *tr*(·) is the trace operation, and m is the dimension of the measurement vector.

In accordance with Equation (15), the approximate posterior PDF of $\lambda_{k}$ can be given by

(26) $$q^{\left(n+1\right)}\left(\lambda_{k}\right)=G\left(\lambda_{k};a_{k}^{\left(n+1\right)},b_{k}^{\left(n+1\right)}\right),$$

where $a_{k}^{\left(n+1\right)}$ and $b_{k}^{\left(n+1\right)}$ denote the shape parameter and rate parameter, respectively. Their values can be given by

(27) $$a_{k}^{\left(n+1\right)}=\frac{1}{2}\left(m+\gamma \right)b_{k}^{\left(n+1\right)}=\frac{1}{2}\left(\gamma +tr\left(A_{k}^{\left(n\right)}E^{\left(n\right)}\left[R_{k}^{-1}\right]\right)\right).$$

Since $\lambda_{k}$ is distributed according to a Gamma distribution, the mathematical expectation of $\lambda_{k}$ can be given by

(28) $$E^{\left(n+1\right)}\left(\lambda_{k}\right)=\frac{a_{k}^{\left(n+1\right)}}{b_{k}^{\left(n+1\right)}}.$$

From the properties of the WD, the mathematical expectation of $R_{k}^{-1}$ can be deduced as follows:

(29) $$E^{\left(n+1\right)}\left(R_{k}^{-1}\right)=\left({\hat{u}}_{k}^{\left(n+1\right)}-m-1\right){\left({\hat{U}}_{k}^{\left(n+1\right)}\right)}^{-1}.$$

The updated measurement noise covariance matrix can be derived from Equations (27) and (28) as follows:

(30) $${\tilde{R}}_{k}^{\left(n+1\right)}={\left(E^{\left(n+1\right)}\left[R_{k}^{-1}\right]\right)}^{-1}/E^{\left(n+1\right)}\left[\lambda_{k}\right].$$

### 3.3. ISRAKF

To further enhance the filtering accuracy of the algorithm, this study introduces the IS robust adaptive Kalman filter (ISRAKF), which effectively eliminates outliers by means of predicting and updating the saturation function. Outliers have the potential to compromise innovation, thereby impacting the efficacy of KF in rectifying state estimation. Therefore, the introduction of saturation functions is used to suppress the divergence of innovation values caused by outlier deviations.

(31) $${\hat{x}}_{k|k}={\hat{x}}_{k|k-1}+K_{k}{\mathrm{sat}}_{\alpha }(e_{k})(z_{k}-H_{k}{\hat{x}}_{k|k-1}),$$

where $e_{k}=z_{k}-H_{k}{\hat{x}}_{k|k-1},$ represents the innovation value, and the saturation function is defined as follows:

(32) $$sat_{\alpha }\left(d\right)=sat_{\sqrt{\alpha_{k}}}\left(d\right)=\left\{\begin{array}{c} -\frac{1}{d}\sqrt{\alpha_{k}},d<-\sqrt{\alpha_{k}}\\ 1\;,\left|d\right|\leq \sqrt{\alpha_{k}}\\ \frac{1}{d}\sqrt{\alpha_{k}},\sqrt{\alpha_{k}}<d, \end{array}\right.$$

where $\alpha_{k}>0$, and $\alpha_{k}$ is the saturation threshold. When the innovation falls within the interval $\left[-{\left(\alpha_{k}\right)}^{1/2},{\left(\alpha_{k}\right)}^{1/2}\right]$, the saturation function becomes a constant (=1), and the PC measurement is fully incorporated into the state estimate. Conversely, if $e_{k}$ is impacted by an outlier and exceeds the anticipated innovation range, the saturation function will reach its limit, thereby mitigating the influence of the outlier.

However, defining a consistent and effective saturation boundary presents significant challenges. If an outlier falls within the saturation range, the effectiveness of the saturation function may be compromised. Additionally, when the update value exceeds the saturation range at a given moment, it can adversely affect the precision of the filtering process. To address these challenges, this paper proposes adaptive strategies for dynamically adjusting the saturation boundary, which can be represented by the following formula:

(33) $$\alpha_{k}=\alpha_{k-1}(1-e^{-\eta_{1}\tau_{k}}),$$

(34) $$\tau_{k}=\xi_{k}e_{k}+\eta_{2}{\left(e_{k}\right)}^{2},$$

where $\tau_{k}>0$, and $\tau_{k}$ denotes the number of connections within the two-layer mechanism. $\xi_{k}$ represents the positive definite coefficient, ensuring that $\tau_{k}$ consistently satisfies the positive condition. $\eta_{1}>0$ and $\eta_{2}>0$. Changing $\eta_{1}$ can adjust the response speed of the saturation boundary to changes in innovation, while changing $\eta_{2}$ can adjust the sensitivity of the saturation boundary to innovation changes. Under normal circumstances, the value of $e_{k}$ will remain within an appropriate range. However, in the presence of outliers during the update process, $e_{k}$ will increase. Simultaneously, as $e_{k}$ increases due to outlier influence, $\alpha_{k}$ will decrease rapidly. This facilitates self-adaptive regulation of the saturation boundary. Consequently, normal measurement values and outlier values can be effectively distinguished from each other, while innovation values affected by outliers can be filtered out. To strengthen the scientific rigor of the proposed method and to provide systematic guidance for selecting the saturation boundary, a formal mathematical analysis is presented in Appendix A.

Combining the estimated results from the preceding section, the iterative ISRAKF prediction process of PC at the (n+1)th iteration is as follows:

(35) $${\hat{x}}_{k|k-1}=\Phi_{k}{\hat{x}}_{k-1|k-1},$$

(36) $$P_{k|k-1}=\Phi_{k}P_{k-1|k-1}\Phi_{k}^{T}+Q_{k-1},$$

where ${\hat{x}}_{k|k-1}$ denotes the “prior,” while $P_{k|k-1}$ represents the error covariance matrix associated with the “prior.” Additionally, ${\hat{x}}_{k-1|k-1}$ and $P_{k-1|k-1}$ denote the “posterior” and “posterior” error covariance matrices of the PC at time k−1. The correction process of ISRAKF can be given by

(37) $$K_{k}^{\left(n+1\right)}=P_{k|k-1}^{\left(n+1\right)}H_{k}^{T}{\left(H_{k}P_{k|k-1}^{\left(n+1\right)}H_{k}^{T}+{\tilde{R}}_{i}^{\left(n+1\right)}\right)}^{-1},$$

(38) $${\hat{x}}_{k|k}^{\left(n+1\right)}={\hat{x}}_{k|k-1}^{\left(n+1\right)}+K_{k}^{\left(n+1\right)}sat_{\sqrt{\alpha_{k}}}(e_{k})\left(z_{k}-H_{k}{\hat{x}}_{k|k-1}\right),$$

(39) $$P_{k|k}^{\left(n+1\right)}=\left(I-K_{k}^{\left(n+1\right)}H_{k}\right)P_{k|k-1}^{\left(n+1\right)},$$

where $K_{k}^{\left(n+1\right)}$ is the Kalman gain, ${\hat{x}}_{k|k}^{\left(n+1\right)}$ is the posterior estimate, and $P_{k|k}^{\left(n+1\right)}$ is the posterior error covariance matrix.

**Figure 1:**
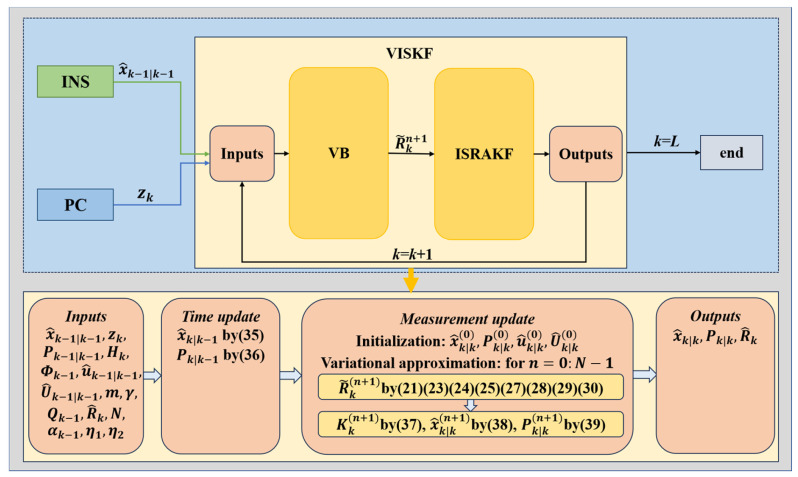
Flowchart depicting the VISKF algorithm.

To facilitate understanding, the workflow is summarized in Algorithm 1, and the algorithm’s structure is illustrated in the flowchart in Figure 1.

By the proposed algorithm, the measurement noise in the INS/PC integrated navigation system is modeled as heavy-tailed noise following the STD, and outliers in the PC are suppressed. Thereby enhancing the performance of the navigation system.
**Algorithm 1: The Proposed VISKF****Inputs:** ${\hat{x}}_{k-1|k-1}$,
$z_{k}$,
$P_{k-1|k-1}$,
$H_{k}$,
$\Phi_{k-1}$,
${\hat{u}}_{k-1|k-1}$,
${\hat{U}}_{k-1|k-1}$,
m,
γ,
$Q_{k-1}$,
${\hat{R}}_{k}$,
N,
$\eta_{1}$$,\eta_{2}$$,\alpha_{k-1}$**Time update:****1: Calculate** ${\hat{x}}_{k|k-1}=\Phi_{k}{\hat{x}}_{k-1|k-1}$**2: Calculate** $P_{k|k-1}=\Phi_{k}P_{k-1|k-1}\Phi_{k}^{T}+Q_{k-1}$**Measurement update:****Initialization:****3:** ${\hat{x}}_{k|k}^{\left(0\right)}={\hat{x}}_{k-1|k-1}$$,P_{k|k}^{\left(0\right)}=P_{k|k-1}$$,{\hat{u}}_{k|k}^{\left(0\right)}=m+1$$,{\hat{U}}_{k|k}^{\left(0\right)}={\hat{R}}_{k}$**For** n=0:N−1**4:** $A_{k}^{\left(n\right)}=(z_{k}-H_{k}{\hat{x}}_{k|k}^{\left(n\right)}){\left(z_{k}-H_{k}{\hat{x}}_{k|k}^{\left(n\right)}\right)}^{T}+H_{k}P_{k|k}^{\left(n\right)}H_{k}^{T}$**5: Calculate** $a_{k}^{\left(n+1\right)},b_{k}^{\left(n+1\right)}$ via (27)**6: Update** $q^{\left(n+1\right)}\left(\lambda_{k}\right)=G\left(\lambda_{k};a_{k}^{\left(n+1\right)},b_{k}^{\left(n+1\right)}\right)$**7: Calculate** $E^{\left(n+1\right)}\left(\lambda_{k}\right)=a_{k}^{\left(n+1\right)}/b_{k}^{\left(n+1\right)}$**8: Calculate** ${\hat{u}}_{k|k}^{\left(n+1\right)}$$,{\hat{U}}_{k|k}^{\left(n+1\right)}$ via (23)
**9: Update** $q^{\left(n+1\right)}\left(R_{k}\right)=IW\left(R_{k};{\hat{u}}_{k|k}^{\left(n+1\right)},{\hat{U}}_{k|k}^{\left(n+1\right)}\right)$**10: Calculate** $E^{\left(n+1\right)}\left(R_{k}^{-1}\right)=\left({\hat{u}}_{k}^{\left(n+1\right)}-m-1\right){\left({\hat{U}}_{k|k}^{\left(n+1\right)}\right)}^{-1}$**11: Calculate** ${\tilde{R}}_{k}^{\left(n+1\right)}={\left(E^{\left(n+1\right)}\left(R_{k}^{-1}\right)\right)}^{-1}/E^{\left(n+1\right)}\left(\lambda_{k}\right)$**12: Update** $\alpha_{k}$$,\tau_{k}$ via (33) and (34)**13:** $K_{k}^{\left(n+1\right)}=P_{k|k-1}^{\left(n+1\right)}H_{k}^{T}{\left(H_{k}P_{k|k-1}^{\left(n+1\right)}H_{k}^{T}+{\tilde{R}}_{i}^{\left(n+1\right)}\right)}^{-1}$**14:** ${\hat{x}}_{k|k}^{\left(n+1\right)}={\hat{x}}_{k|k-1}^{\left(n+1\right)}+K_{k}^{\left(n+1\right)}sat_{\sqrt{\alpha_{k}}}(e_{k})\left(z_{k}-H_{k}{\hat{x}}_{k|k-1}\right)$**15:** $P_{k|k}^{\left(n+1\right)}=\left(I-K_{k}^{\left(n+1\right)}H_{k}\right)P_{k|k-1}^{\left(n+1\right)}$**end for****16:** ${\hat{x}}_{k|k}\leftarrow {\hat{x}}_{k|k}^{\left(N\right)}$$,P_{k|k}\leftarrow P_{k|k}^{\left(N\right)}$$,{\hat{R}}_{k}\leftarrow {\tilde{R}}_{k}^{\left(N\right)}$**Outputs:** ${\hat{x}}_{k|k}$$,P_{k|k}$$,{\hat{R}}_{k}$

## 4. Empirical Validation and Statistical Analysis

### 4.1. Experimental Equipment

**Figure 2:**
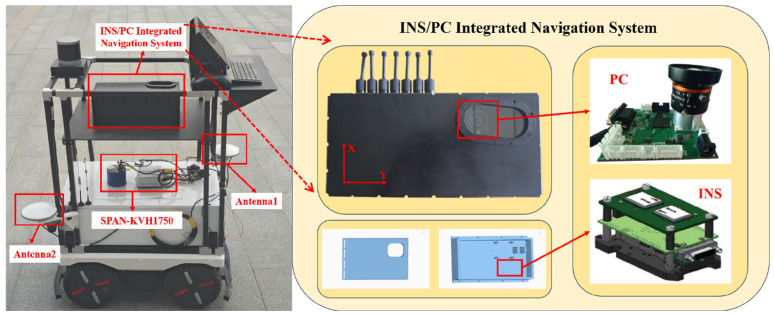
The configuration of INS/PC-integrated navigation system and the unmanned navigation test platform.

To validate the efficacy of the proposed algorithm, static and dynamic experiments were conducted on an unmanned navigation test platform situated on a campus safety road, yielding a substantial volume of reliable data. The SPAN-KVH1750 system (Ronghui Positioning Technology Co., Ltd., Beijing, China) is employed as the heading reference. The configuration of the unmanned navigation test platform and the INS/PC integrated navigation system is illustrated in Figure 2, while sensor parameters are detailed in Table 1. The experimental setup utilizes a self-designed polarization compass, which employs the ZYNQ7020 chip (Shenzhen Wanchuang Xinda Electronics Co., Ltd., Shenzhen, China) as the computational core and the Sony IMAX250MZR CMOS (Shenzhen Wanchuang Xinda Electronics Co., Ltd., Shenzhen, China) as the polarization image sensor. To enhance computational efficiency, the polarization compass skips graphical processing and relies solely on the light intensity information from a central 40 × 40 region to calculate the polarization angle. The solar azimuth is computed using pre-input values of latitude, longitude, and time, with time updates provided by the internal chip clock.

### 4.2. Experimental Investigation of the Dynamic Condition

To comprehensively evaluate the proposed algorithm, ablation experiments and comparative experiments with comparable algorithms were conducted under both static and dynamic conditions. Among the control groups in the ablation experiments are AKF; ISRAKF; and VBAKF. The details of the method parameters for the ablation experiment are shown in Table 2.

The similar target algorithms included in the comparative experiment are described below.

Huber’s M-estimation-Based Robust Adaptive Kalman Filter (Huber-based RAKF): The algorithm adopts Huber’s M-estimation paradigm. During the measurement-update step, an iterative scheme is constructed in which Huber’s M-estimator is employed to rescale—effectively inflate—the prior state-estimation covariance via a weighting matrix. This inflation mitigates the influence of process uncertainties induced by abrupt maneuvers. Iteration proceeds until all elements of the weight matrix converge to unity, a criterion that also minimizes computational effort [15].

Maximum Correntropy Criterion Kalman Filter (MCCKF): To enhance the robustness of the KF in non-Gaussian environments, the maximum correntropy Kalman filter (MCKF) replaces the conventional minimum-mean-square-error (MMSE) criterion with the maximum correntropy criterion (MCC) and introduces a fixed-point iteration scheme for its solution. By substituting correntropy for the mean-square-error cost and employing fixed-point iterations in the update step, the algorithm transmutes the traditional KF into a variant exhibiting markedly improved robustness against impulsive noise [17].

Among these algorithms, AKF serves as a baseline control group for evaluating the performance improvement achieved by various algorithms. Meanwhile, AKF, ISRAKF and VBAKF, along with the proposed algorithm, are employed as control groups in ablation experiments to investigate the impact of each module of the proposed algorithm. Huber-based RAKF and MCCKF serve as comparative benchmarks to evaluate the optimization impact of the proposed algorithm in comparison with other similar target algorithms.

The main challenges encountered by PC involve image degradation, including loss, distortion, and blurring, which are primarily caused by occlusion, vehicle turning, and high maneuverability. These complexities may result in outliers from PC. Obstruction to the PC may result in the loss of atmospheric polarization information captured by the PC. This information loss introduces an anomaly in solving the polarization angle, which in turn prevents the PC from outputting the correct heading angle. When shooting on a rapidly maneuvered carrier, the PC can theoretically remain relatively still in the current scene as long as the exposure time is sufficiently short. This is because shorter exposure times reduce motion blur and enhance image sharpness. However, in practice, the exposure time of the camera cannot be set too short due to specific brightness requirements for polarization heading resolution. If the exposure time is excessively short and insufficient light reaches the sensor, it will significantly increase calculated heading angle errors and may even cause algorithm failure. Therefore, solely reducing exposure time and adjusting sensor gain are not feasible approaches to mitigate high mobility’s adverse effects on PC performance. In fact, during actual motion, the image is directly captured on the CMOS sensor, resulting in a blurred photograph with trailing effects. Consequently, PC will inevitably produce outliers. Therefore, to optimize the overall performance of the integrated navigation system, it is imperative to mitigate these adverse effects while simultaneously enhancing navigation accuracy and stability. To assess the efficacy of the proposed algorithm in addressing the aforementioned issues, additional tests were conducted using a vehicle to execute high-speed maneuvers and turns for the INS/PC system. Specifically, high-speed movements and multiple turns were performed on the PC for a specific duration, as outlined in Table 3.

To evaluate the efficacy of the proposed algorithm in dynamic scenarios, a 1200 s dynamic experiment was conducted on 24 June 2025, at 4:30 p.m. under clear weather conditions.

**Figure 3:**
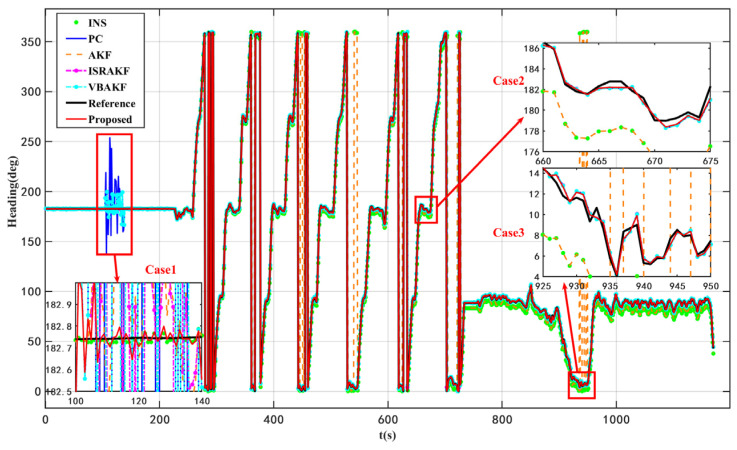
Headings of ablation dynamic experiments.

**Figure 4:**
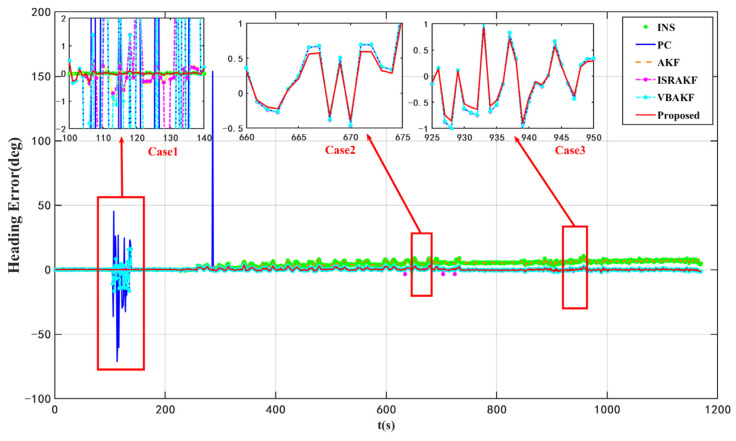
Heading errors of ablation dynamic experiments.

According to the results presented in Figure 3 and Figure 4 as well as Table 4, the proposed VISKF algorithm demonstrates effective suppression of outliers in PC during vehicle turning and high-speed movement, while also exhibiting high maneuverability.

The proposed algorithm demonstrates an 84.45% improvement in accuracy compared to the AKF algorithm. Furthermore, it is evident that the various modules of the VISKF algorithm maintain their effectiveness under dynamic conditions.

**Figure 5:**
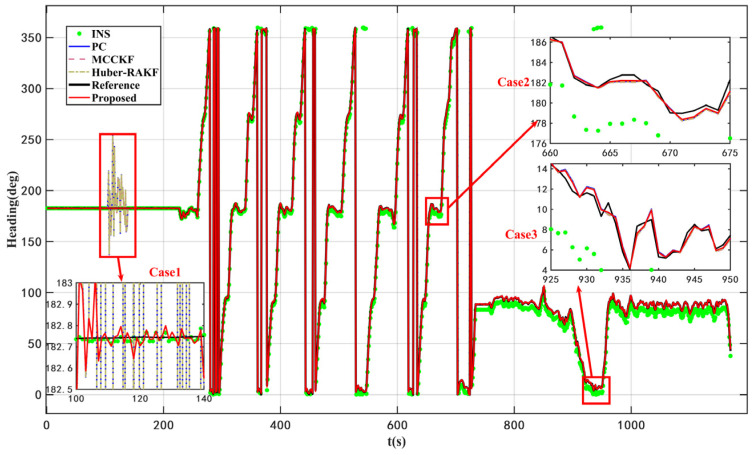
Headings of dynamic experiments.

**Figure 6:**
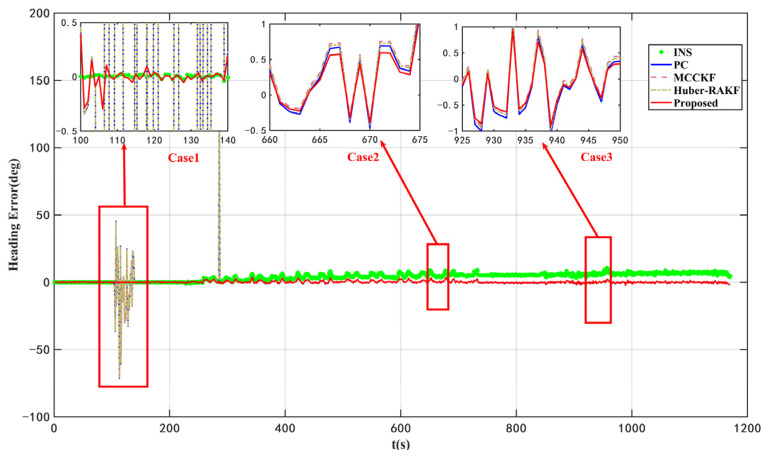
Heading errors of dynamic experiments.

As depicted in Figure 5 and Figure 6, as well as Table 5, the proposed VISKF algorithm exhibits superior performance in enhancing navigation accuracy and system robustness compared to alternative algorithms, thereby significantly outperforming them.

The complexity of time-varying noise encountered by PC in dynamic conditions is primarily attributed to environmental factors, sensor characteristics, and variations in system state. These elements collectively contribute to the temporal variability of noise characteristics and distributions. However, the Huber-based RAKF algorithm presumes the reliability of external measurements and therefore lacks the capability to simultaneously address the coexistence of measurement and process uncertainties. Furthermore, the Huber-based RAKF algorithm fails to timely adjust its parameters to accommodate new noise characteristics, potentially leading to inaccurate estimation outcomes. MCCKF is intrinsically limited by the Gaussian kernel’s local approximation capacity when confronted with extremely heavy-tailed or unknown noise distributions; it can only mediate between over-smoothing and over-sensitivity through a bandwidth parameter that must be set a priori. In contrast, the proposed algorithm adopts a heavy-tailed Student’s t noise model and leverages online variational Bayes to directly estimate the time-varying scale matrix. This formulation preserves higher-order statistical information while eliminating the need for an exact, pre-specified covariance. Consequently, VISKF demonstrates markedly superior adaptability and robustness in environments characterized by severe outliers and complex, non-Gaussian noise structures.

In the above field experiments, the degree of freedom parameter and the number of iteration in the proposed method are chosen as γ=5 and N=10. and the initial value of the saturation boundary is chosen as $\alpha_{0}=9$. The other parameters in the innovation saturation mechanism are set as $\eta_{1}=0.01$, $\eta_{2}=0.01$ and $\xi_{k}=0.5\times abs(e_{k})/e_{k}$.

The setting parameters are set as follows:

(40) $${\hat{x}}_{0\left|0\right.}=zeros\left(15,0\right),$$

(41) $$P_{0\left|0\right.}=diag{\left\{\left[\nabla \phi ;\nabla V;\nabla P;\omega^{b};f^{b}\right]\right\}}^{2},$$

(42) $$Q_{0}=diag{\left\{\left[\sigma_{g}^{b};\sigma_{a}^{b};zeros\left(9,1\right)\right]\right\}}^{2},$$

(43) $$R_{0}=diag{\left(\delta \psi \right)}^{2},$$

where

(44) $$\left\{\begin{array}{l} \nabla \phi =\left[0.18{}^{\circ},0.04{}^{\circ},-0.01{}^{\circ}\right]\\ \nabla V=\left[0.1m/s,0.1m/s,0.1m/s\right]\\ \nabla P=\left[0.5m,0.5m,0.5m\right]\\ \omega^{b}=\left[0.02{}^{\circ}/h,0.02{}^{\circ}/h,0.05{}^{\circ}/h,\right]\\ f^{b}=\left[2000\mu g,1400\mu g,500\mu g\right]\\ \sigma_{g}^{b}=\left[0.028{}^{\circ}/h,0.028{}^{\circ}/h,0.028{}^{\circ}/h\right]\\ \sigma_{a}^{b}=\left[5\times 10^{-3}g,5\times 10^{-3}g,5\times 10^{-3}g\right]. \end{array}\right.$$

The variational Bayesian method and innovation saturation mechanism introduce additional computational complexity. Consequently, this paper conducts a real-time performance analysis of AKF, ISRAKF, and VBAKF. The single-step implementation CPU time of various algorithms is given by Table 6. The experiments were performed on an AMD Ryzen 9 7945HX CPU (Lenovo Limited, Beijing, China). CPU time is crucial in real-time systems. If CPU time is excessively prolonged, real-time requirements may not be satisfied. Through the reasonable parameter selection, the proposed algorithm can meet the real-time requirements of the INS/PC integrated navigation system.

## 5. Conclusions

In this paper, an improved adaptive Kalman filter is proposed to solve the problem of measurement outliers caused by PC in occlusion environments and during turns and high-speed movements in the INS/PC integrated navigation system. The proposed VISKF algorithm uses the STD to better approximate the posterior distribution of PC measurement noise, thereby overcoming the measurement anomalies faced by PC in complex environments when performing navigation information fusion with INS. In addition, a saturation function is introduced to suppress the divergence of the update vector, further eliminating outliers. The experimental results show that the algorithm has a very precise and sensitive response to the noise and outliers of PC. In future work, additional experiments will be conducted in more diverse and challenging scenarios, such as underwater and aerial environments. In addition, the complex meteorological conditions are also an important factor affecting the accuracy of polarization orientation. Next, we will also conduct experiments under various weather conditions (such as cloudy, rainy, etc.) to further verify the adaptability of the proposed algorithm in a more comprehensive manner. Furthermore, research into more flexible and general noise models capable of handling a wider range of noise types will be pursued. Additionally, exploring the application of noise models to different fields, including autonomous driving, UAV navigation, and robot navigation, will be important directions for future research.

## Figures and Tables

**Table 1:** Table of sensor parameters.

Sensors	Parameters	Value
SPAN-KVH1750	Heading accuracy (RMS)	0.035°
	Output frequency	100 Hz
INS	Heading accuracy (RMS)	0.15°/m
	Output frequency	100 Hz
PC	Static heading accuracy (RMS)	0.3°
	Dynamic heading accuracy (RMS)	0.5°
	Output frequency	15 Hz

**Table 2:** Detailed ablation method configuration.

Sensors	Adaptive Mechanism	IS Mechanism	Improved VB
AKF	√	×	×
ISRAKF	√	√	×
VBAKF	√	×	√
**Proposed**	**√**	**√**	**√**

**Table 3:** Experimental procedures for dynamic conditions.

Case	Detailed Operation
Case1	Traverse the shaded road section.
Case2	Continuously making multiple turns and randomly select one.
Case3	The carrier exhibits high mobility and any segment of it can be selectively utilized.

**Table 4:** Max, Min, Mean, and RMS of ablation experiment heading angle error for various methods (unit: °).

Method	Max	Min	Mean	RMS	Improve
INS	10.66	–1.07	3.98	4.78	/
PC	153.90	–39.85	0.25	5.65	/
AKF	10.64	–1.07	3.97	4.76	/
ISRAKF	9.16	–3.60	0.24	1.10	76.89%
VBAKF	16.76	–13.71	0.21	1.56	67.23%
**Proposed**	**3.59**	**–** **2.09**	**0.18**	**0.74**	**84.45%**

**Table 5:** Max, Min, Mean, and RMS of experiment heading angle error for various methods (unit: °).

Method	Max	Min	Mean	RMS	Improve
AKF	10.64	−1.07	3.97	4.76	/
Huber-based RAKF	152.40	−39.46	0.29	3.60	24.37%
MMCKF	151.40	−39.20	0.31	2.56	46.21%
**Proposed**	**3.59**	**−2.09**	**0.18**	**0.74**	**84.45%**

**Table 6:** Single-step runtime of various algorithms (unit: s).

	AKF	ISRAKF	VBAKF	Proposed
**CPU Time**	2.47 × 10^−6^	2.59 × 10^−6^	2.83 × 10^−5^	**2.84 × 10^−5^**

## Data Availability

The datasets presented in this article are not readily available because the data are part of an ongoing study.

## Appendix A

The bound analysis on the adaptive saturation mechanism is described below.

Without loss of generality, consider discrete-time systems

(A1) $$\left\{\begin{array}{l} x_{k+1}=\Phi_{k}x_{k}+w_{k}\\ z_{k}=H_{k}x_{k}+n_{k}+Dd_{k} \end{array}\right.,$$

where $n_{k}$ denotes noise, $d_{k}$ denotes the bounded outliers, and D denotes the relationship between $d_{k}$ and $z_{k}$, which is assumed to be unknown.

The state prediction error is defined as ${\tilde{x}}_{\left.k\right|k}=x_{k}-{\hat{x}}_{\left.k\right|k}$, and its recurrence relation is given by

(A2) $${\tilde{x}}_{\left.k\right|k}=\left(I-K_{k}sat_{\sqrt{\alpha_{k}}}\left(e_{k}\right)\right){\tilde{x}}_{\left.k\right|k-1}-K_{k}sat_{\sqrt{\alpha_{k}}}\left(e_{k}\right)\left(Dd_{k}+n_{k}\right).$$

The measurement noise $n_{k}$ that excludes outliers can be absorbed by $R_{k}$, so it is reasonable to assume that $n_{k}$ can be ignored, and then Equation (A2) can be simplified as follows:

(A3) $${\tilde{x}}_{\left.k\right|k}=\left(I-K_{k}sat_{\sqrt{\alpha_{k}}}\left(e_{k}\right)\right){\tilde{x}}_{\left.k\right|k-1}-K_{k}sat_{\sqrt{\alpha_{k}}}\left(e_{k}\right)Dd_{k}.$$

The Lyapunov function is constructed as follows:

(A4) $$L_{k}={\tilde{x}}_{\left.k\right|k}^{T}P_{\left.k\right|k}^{-1}{\tilde{x}}_{\left.k\right|k}+\alpha_{k}.$$

Next, a recursive analysis of the Lyapunov function is conducted to calculate $L_{k+1}-L_{k}$.

(A5) $$L_{k+1}-L_{k}={\tilde{x}}_{\left.k+1\right|k}^{T}P_{\left.k+1\right|k}^{-1}{\tilde{x}}_{\left.k+1\right|k}-{\tilde{x}}_{\left.k\right|k}^{T}P_{\left.k\right|k}^{-1}{\tilde{x}}_{\left.k\right|k}+\left(\alpha_{k+1}-\alpha_{k}\right).$$

In the system of this paper, the process noise $w_{k}$ is bounded and satisfies $\left\|w_{k}\right\|\leq w$; then,

(A6) $${\tilde{x}}_{\left.k+1\right|k}^{T}P_{\left.k+1\right|k}^{-1}{\tilde{x}}_{\left.k+1\right|k}\leq {\tilde{x}}_{\left.k\right|k}^{T}\Phi_{k}^{T}P_{\left.k+1\right|k}^{-1}\Phi_{k}{\tilde{x}}_{\left.k\right|k}+\varepsilon w^{2},$$

where ε is a constant related to $\Phi_{k}$ and $P_{\left.k+1\right|k}^{-1}$.

According to Equation (36), since $Q_{k}$ is a positive definite matrix, if $Q_{k}$ is ignored, its inverse matrix can be approximated as

(A7) $$P_{\left.k+1\right|k}^{-1}\leq \Phi_{k}^{-T}P_{\left.k\right|k}^{-1}\Phi_{k}^{-1}.$$

It can be further obtained that

(A8) $${\tilde{x}}_{\left.k+1\right|k}^{T}P_{\left.k+1\right|k}^{-1}{\tilde{x}}_{\left.k+1\right|k}\leq {\tilde{x}}_{\left.k\right|k}^{T}P_{\left.k\right|k}^{-1}{\tilde{x}}_{\left.k\right|k}+\varepsilon w^{2}.$$

Combining Equations (A5) and (A8) yields

(A9) $$\begin{array}{c} L_{k+1}-L_{k}\leq \left({\tilde{x}}_{\left.k\right|k}^{T}P_{\left.k\right|k}^{-1}{\tilde{x}}_{\left.k\right|k}+\varepsilon w^{2}\right)-{\tilde{x}}_{\left.k\right|k}^{T}P_{\left.k\right|k}^{-1}{\tilde{x}}_{\left.k\right|k}+\left(\alpha_{k+1}-\alpha_{k}\right)\\ \;=\varepsilon w^{2}+\left(\alpha_{k+1}-\alpha_{k}\right). \end{array}$$

According to Equations (33) and (34) and $\eta_{1}>0$,$\tau_{k}>0$, it follows

(A10) $$\alpha_{k+1}-\alpha_{k}=-\alpha_{k}e^{-\eta_{1}\tau_{k}}\leq -\alpha_{k}\left(1-\eta_{1}\tau_{k}\right).$$

In order not to change the original positive and negative properties, the parameters $\eta_{1}$ and $\tau_{k}$ should be chosen such that $1-\eta_{1}\tau_{k}\geq \beta >0$; then,

(A11) $$L_{k+1}-L_{k}\leq \varepsilon w^{2}-\beta \alpha_{k}.$$

Recursively sum to get

(A12) $$L_{k}\leq L_{0}-\beta \sum_{i=0}^{k-1}\alpha_{i}+\varepsilon w^{2}k.$$

According to Equation (A4), the lower bound property of the quadratic form can be obtained

(A13) $$L_{k}\geq \lambda_{\min}\left(P_{\left.k\right|k}^{-1}\right){\left\|{\tilde{x}}_{\left.k\right|k}\right\|}^{2}+\alpha_{k},$$

where $\lambda_{\min}\left(P_{\left.k\right|k}^{-1}\right)$ is the smallest eigenvalue of the matrix.

When k→∞, the system tends to steady state, the covariance matrix converges to the steady state value $P_{\infty }^{-1}$, its smallest eigenvalue is constant $\lambda_{\min}\left(P_{\infty }^{-1}\right)$, and the adaptation parameter $\alpha_{k}$ tends to the steady state value $\alpha_{\infty }$.

In this case, according to Equation (A11) and $L_{k+1}=L_{k}=L_{\infty }$, $\alpha_{\infty }$ satisfies

(A14) $$0\leq -\beta \alpha_{\infty }+\varepsilon w^{2}\Rightarrow \alpha_{\infty }\leq \frac{\varepsilon w^{2}}{\beta }.$$

Combining Equations (A12) and (A13) yields

(A15) $$\lambda_{\min}\left(P_{\infty }^{-1}\right){\left\|{\tilde{x}}_{k}\right\|}^{2}\leq L_{k}-\alpha_{k}\leq L_{0}+\varepsilon w^{2}k-\beta \sum_{i=0}^{k-1}\alpha_{i}-\alpha_{k}.$$

To make the right-hand side bounded, it is necessary that the cumulative effect $\varepsilon w^{2}k$ of the process noise (that grows linearly) is canceled out by the cumulative decay $\beta \sum \alpha_{i}$ of the adaptive parameters, i.e.,

(A16) $$\sum_{i=0}^{k-1}\alpha_{i}\geq \frac{\varepsilon w^{2}}{\beta }k+Constant.$$

By the gradual behavior of the accumulation sum, $\underset{k\to \infty }{\lim}\sum_{i=0}^{k-1}\alpha_{i}\approx \alpha_{\infty }k$ can be obtained; then, by substituting it in Equation (A16), it gives

(A17) $$\alpha_{\infty }k\geq \frac{\varepsilon w^{2}}{\beta }k-Constant.$$

When k→∞, the influence of the constant term can be ignored, and thus

(A18) $$\alpha_{\infty }\geq \frac{\varepsilon w^{2}}{\beta }.$$

By Equations (A14) and (A18), $\alpha_{\infty }=\varepsilon w^{2}/\beta$ can be obtained. Substituting $\alpha_{\infty }$ and $\sum_{i=0}^{k-1}\alpha_{i}\approx \alpha_{\infty }k$ into Equation (A15), the following can be obtained:

(A19) $$\lambda_{\min}\left(P_{\infty }^{-1}\right){\left\|{\tilde{x}}_{k}\right\|}^{2}\leq L_{0}+\varepsilon w^{2}k-\beta \left(\frac{\varepsilon w^{2}}{\beta }\right)k-\frac{\varepsilon w^{2}}{\beta }=L_{0}-\frac{\varepsilon w^{2}}{\beta }.$$

After simplification, the limiting supremely bound of the state prediction error can be obtained as

(A20) $$\underset{k\to \infty }{\lim}\sup\left\|{\tilde{x}}_{k}\right\|\leq \sqrt{\frac{\beta L_{0}-\varepsilon w^{2}}{\beta \lambda_{\min}\left(P_{\infty }^{-1}\right)}},$$

where $L_{0}$ needs to satisfy $L_{0}\geq \varepsilon w^{2}/\beta$. The supremely bound limit of the prediction error indicates that the system can maintain the stability of the state estimation in the presence of long-term external disturbances or internal uncertainties. In addition, it also guides the selection of key parameters in engineering applications. Choosing the value of $\eta_{1}$ follows from $\eta_{1}>0$ and $0<\eta_{1}\tau_{k}<1$, and the value of $\eta_{1}$ can be scientifically selected by combining the error bound of PC. When selecting other parameters, the constraint condition of bounded state prediction error should be combined with $\eta_{1}$.
